# An ultra‐high‐density genetic map provides insights into genome synteny, recombination landscape and taproot skin colour in radish (*Raphanus sativus* L.)

**DOI:** 10.1111/pbi.13195

**Published:** 2019-07-04

**Authors:** Xiaobo Luo, Liang Xu, Yan Wang, Junhui Dong, Yinglong Chen, Mingjia Tang, Lianxue Fan, Yuelin Zhu, Liwang Liu

**Affiliations:** ^1^ National Key Laboratory of Crop Genetics and Germplasm Enhancement Key Laboratory of Horticultural Crop Biology and Genetic Improvement (East China) of MOA, College of Horticulture Nanjing Agricultural University Nanjing China; ^2^ Guizhou Institute of Biotechnology Guizhou Academy of Agricultural Sciences Guiyang China; ^3^ The UWA Institute of Agriculture, and School of Agriculture and Environment The University of Western Australia Perth WA Australia

**Keywords:** radish, single nucleotide polymorphism (SNP), genetic map, recombination, root skin colour

## Abstract

High‐density genetic map is a valuable tool for exploring novel genomic information, quantitative trait locus (QTL) mapping and gene discovery of economically agronomic traits in plant species. However, high‐resolution genetic map applied to tag QTLs associated with important traits and to investigate genomic features underlying recombination landscape in radish (*Raphanus sativus*) remains largely unexplored. In this study, an ultra‐high‐density genetic map with 378 738 SNPs covering 1306.8 cM in nine radish linkage groups (LGs) was developed by a whole‐genome sequencing‐based approach. A total of 18 QTLs for 11 horticulture traits were detected. The map‐based cloning data indicated that the R2R3‐MYB transcription factor *RsMYB90* was a crucial candidate gene determining the taproot skin colour. Comparative genomics analysis among radish, *Brassica rapa* and *B. oleracea* genome revealed several genomic rearrangements existed in the radish genome. The highly uneven distribution of recombination was observed across the nine radish chromosomes. Totally, 504 recombination hot regions (RHRs) were enriched near gene promoters and terminators. The recombination rate in RHRs was positively correlated with the density of SNPs and gene, and GC content, respectively. Functional annotation indicated that genes within RHRs were mainly involved in metabolic process and binding. Three QTLs for three traits were found in the RHRs. The results provide novel insights into the radish genome evolution and recombination landscape, and facilitate the development of effective strategies for molecular breeding by targeting and dissecting important traits in radish.

## Introduction

High‐density genetic maps are crucial for genetic and genomic studies, such as mapping quantitative trait loci (QTLs) associated with important agronomic traits in crops. QTL mapping resolution is dependent on marker density and genetic population size (Xu *et al*., 2013). High‐throughput resequencing has been proposed to facilitate the development of markers, genotyping, and increase the marker density of genetic maps (Cao *et al*., 2011; Hu *et al*., 2017). A sliding window approach combining with whole‐genome resequencing (WGRS) data was employed for genotype calling and recombination breakpoint determination (Huang *et al*., 2009; Xu *et al*., 2013). It has been widely applied to construct the recombinant bin map based on the SNP markers and identify important QTLs or genes in various important crops including rice (Gao *et al*., 2013), *Brassica rapa* (Yu *et al*., 2013) and soybean (Lu *et al*., 2017). A bin map was constructed by genotyping a recombinant inbred line (RIL) population and identified the candidate genes involved in yield‐associated trait in rice (Gao *et al*., 2013). In soybean, the high‐density maps generated from WGRS were also effectively applied in mapping a QTL harbouring candidate genes *PP2C* for seed weight (Lu *et al*., 2017). Using high‐density map based on sequencing‐based genotyping method, these findings provide solid basis for QTL mapping associates with important traits.

Anthocyanins, an important flavonoid compound, are responsible for the different colours of varying vegetables, flowers and fruits (Jaakola, 2013; Yuan *et al*., 2009, 2016). Anthocyanins possess potent antioxidant capacities, providing a variety of health‐promoting benefits including protection against cardiovascular disease, obesity and diabetes (He and Giusti, 2010; Nabavi *et al*., 2015). Increasing number of studies showed that anthocyanins play important roles in attracting pollinators and seed dispersers, as well as protecting high light stress and pathogen attack (Allan *et al*., 2008; Grotewold, 2006; Yamagishi *et al*., 2014). The biosynthesis of anthocyanin is mainly regulated by the expression of structural genes at the transcriptional level in plants. In apple, the *MdMYB10* alleles were shown to be correlated with anthocyanin accumulation and were higher in red‐fruited than in green‐fruited cultivars (Ban *et al*., 2007; Takos *et al*., 2006). The *PavMYB10.1* alleles regulate anthocyanin accumulation in sweet cherry skin (Jin *et al*., 2016). It is well known that MYB, basic helix–loop–helix (bHLH) transcription factors (TFs) and a WD‐repeat (WDR) protein (MBW complex) can coordinately activate the late steps towards anthocyanin and proanthocyanidin pathway, which binds the promoters of structural genes to induce their transcription (Koes *et al*., 2005; Xu *et al*., 2015b). The QTL underlying red pigmentation of radish taproot was mapped on LG11 at positions 29.1 and 56.1 cM, respectively (Tsuro *et al*., 2008). Nevertheless, the molecular genetic mechanism underlying taproot skin colour in radish remained poorly understood.

Meiotic recombination is an essential biological process for the generation of new combinations of alleles and plays an important role in genomic evolution (Si *et al*., 2015). Recombination contributes to influence the plant breeding programmes by affecting the efficiency of natural selection (Silva‐Junior and Grattapaglia, 2015). High‐density genetic map, as a classic tool to study recombination, can be measured through comparing genetic and physical distances (cM/Mb) on the genome. The distribution of recombination is not uniformly distributed along the genome, and the location of recombination is more likely to occur in gene transcriptional start sites (TSSs) and transcriptional termination sites (TTSs) (Choi *et al*., 2013; Pan *et al*., 2016). The numerous genomic features can directly influence the recombination rates, such as GC content, DNA polymorphism, density of gene and transposable elements (Auton *et al*., 2012; Hunter *et al*., 2016). The recombination events occur more frequently than in other regions were defined as recombination hotspots. It was shown that the *CpCYC‐b* gene has been identified to be associated with fruit flesh colour and located in the recombination hotspot region (Blas *et al*., 2010). The intraspecific variation for recombination was observed in different maize populations, and intragenic recombination significantly correlates with gene expression and phenotypic variation by construction of high‐density genetic map, suggesting the potential roles for recombination in plant phenotypic variation (Bauer *et al*., 2013; Pan *et al*., 2016). To date, little information is known about the genome‐wide recombination and genomic feature correlation with recombination rate in radish.

Radish (*Raphanus sativus* L., 2n = 2*x* = 18) is an important root vegetable crop of Brassicaceae family. Comprehensive identification of key genes associated with root‐related traits is the prerequisite for exploitation in radish genetic improvement. In the past decade, several radish genetic maps had been developed basing on traditional molecular markers and used for QTL analysis of resistance against beet cyst nematode, cadmium accumulation and 4‐methylthio‐3‐butenyl glucosinolate contents in radish (Budahn *et al.*, 2009; Xu *et al.*, 2012; Zou *et al.*, 2013). A genetic map with 258 SSR markers was applied to detect QTLs for 11 morphological traits (Yu *et al.*, 2016). Recently, a genetic map was constructed with 2637 single nucleotide polymorphisms (SNPs) by resequencing 93 F_2_ individuals in radish (Mun *et al.*, 2015). However, these maps with small populations or low‐density markers are still not effective for fine mapping of QTLs/genes and marker‐assisted breeding (MAS) in radish.

In this study, an ultra‐high‐density linkage map was constructed by resequencing a F_2_ population derived from a cross between ‘NAU‐LB’ and ‘NAU‐YH’. A large number of QTLs were identified for some important traits basing on the high‐density genetic linkage map. The candidate gene associated with taproot skin colour was isolated using QTL mapping and map‐based cloning techniques. Furthermore, the high‐density genetic map was used to perform comparative genomics analysis and explore the genome‐wide patterns of recombination. These results could provide valuable resource for enhancing our understanding of genome evolution and recombination landscape, and facilitate effective fine mapping and isolating of quantitative trait genes controlling important traits in radish.

## Results

### Population sequencing and linkage map construction in radish

**Figure 1:**
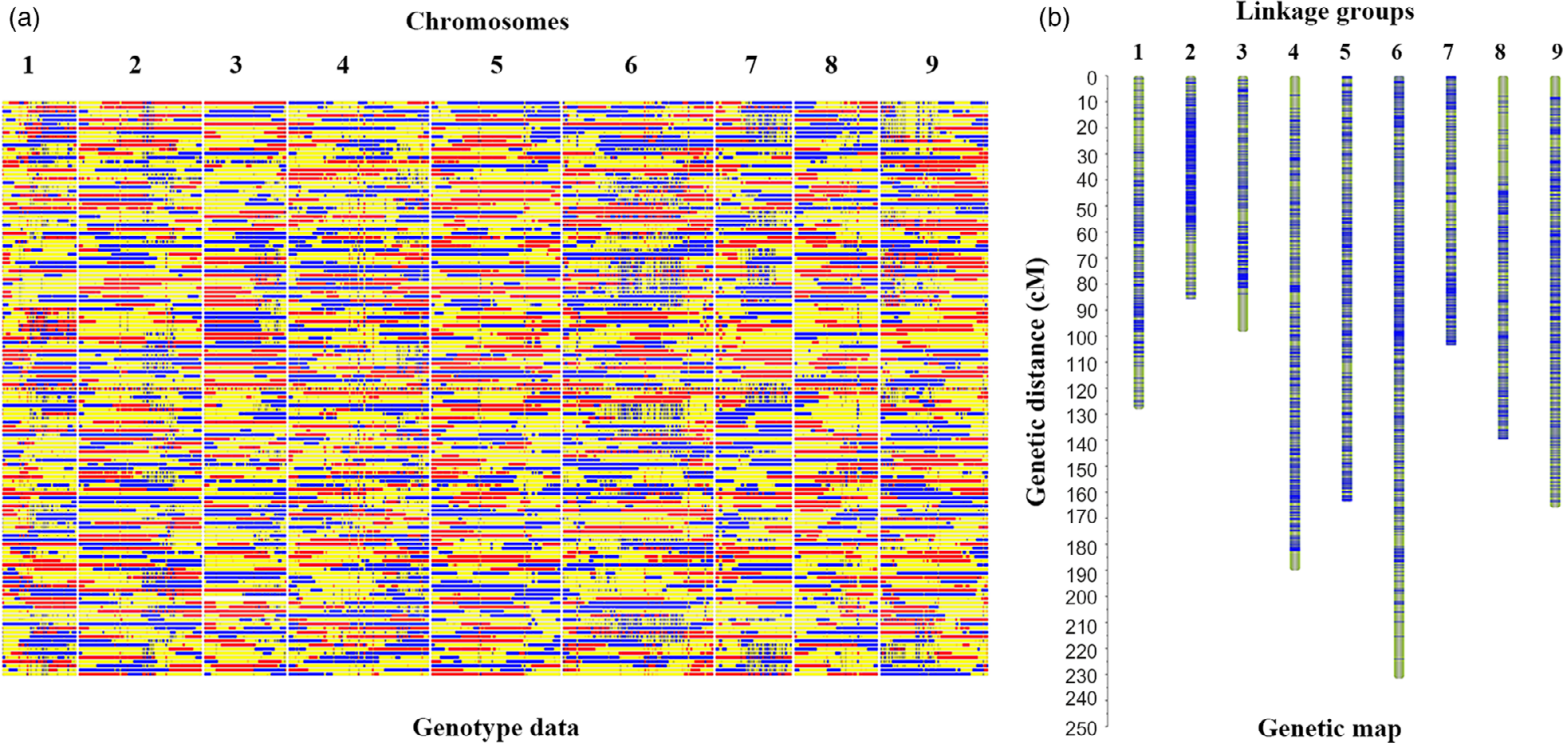
Recombination bin map (a) and genetic map (b) of 137 F_2_ individuals.

To construct a high‐resolution genetic linkage map in radish, the whole‐genome resequencing of 137 F_2_ individuals together with the parental lines was performed on Illumina HiSeq^TM^ 2500 platform. In total, 7.4 Gb (17.3‐fold genome coverage) and 5.6 Gb (12.7‐fold genome coverage) clean reads were generated for the ‘NAU‐LB’ and ‘NAU‐YH’, respectively (Table S1). A total of 403 Gb clean reads were generated for the 137 F_2_ individuals, with an average of 7.2 depth for each individual. The clean reads were mapped against the newly available radish reference genome. A total of homozygous 821 217 SNPs were detected between the two parental lines. After filtering SNPs by the genotyping criteria, 411 891 SNPs were retained to generate bin markers among F_2_ population. The SNP with significant segregation distortion (*P *<* *0.001) was excluded for further analysis. A modified sliding window approach was performed to determine recombinant breakpoints for the F_2_ individuals. The adjacent bins with the same genotype were merged as a bin, and a high‐density genetic map was constructed with 2852 recombination bin markers (378 738 SNPs) (Figures 1 and S1). The total genetic distance of the bin map was 1306.8 cM, with an average distance of 0.46 cM between adjacent bin markers (Tables 1 and S2). These bin markers ranged from 85.73 to 231.51 kb, with an average physical length of 120.91 kb. According to the bin marker locations on the radish reference genome, a total of 40 802 genes were found in 2852 bins, ranging from 3285 to 6317 genes in each chromosome.

**Table 1:** Summary of the nine linkage groups in radish

Linkage group	Number of SNP	Number of bin	Genetic distance (cM)	Average distance (cM)	Average length of bin (kb)	Number of gene	Recombination rate (cM/Mb)
LG01	28 947	217	128.16	0.59	128.16	3285	4.87
LG02	57 202	372	85.73	0.23	85.73	5229	1.96
LG03	43 429	231	98.37	0.43	98.37	3345	3.38
LG04	38 657	400	190.06	0.48	190.06	5992	3.80
LG05	61 262	374	163.63	0.44	163.63	5886	3.56
LG06	67 894	453	231.51	0.51	231.51	6317	4.32
LG07	25 133	233	103.58	0.44	103.58	3293	3.81
LG08	21 230	235	139.78	0.59	139.78	3302	4.71
LG09	34 984	337	165.98	0.49	165.98	4153	4.33
Total	378 738	2852	1306.80	0.46	120.91	40802	3.80

In total, 335 (11.75%) and 270 (9.47%) bin markers exhibited significant segregation distortion at the *P *<* *0.005 and *P *<* *0.001, respectively (Figure S2, Table S3). Among the distorted markers, 316 (93%) bins skewed to the paternal alleles (‘NAU‐YH’), while 11 (3.28%) bins skewed to the maternal alleles (‘NAU‐LB’) and 8 (2.39%) segregated in favour of the heterozygous alleles. A distorted region with more than three adjacent loci was considered as a segregation distortion region (SDR). A total of 19 SDRs were identified on seven linkage groups (LGs) with LG6 (7 SDRs) having the largest number of SDRs (Table S4). Since the segregation distortion has less effect on mapping accuracy and could increase the coverage of the linkage groups (Bartholomé *et al*., 2015), the distorted bin markers were retained in the linkage maps.

### QTL mapping of important horticulture traits in radish

Phenotypic trait data in F_2_ populations exhibited approximately normal distributions (Figure S3). The negative and positive correlations between each pair among the ten traits were evaluated (Table S5). A total of 17 QTLs were detected for ten traits in the bin map using MQM analysis, seven of which were mapped onto the same LG with the reported QTL, providing support for the accuracy of this map (Yu *et al.*, 2016) (Table S6). All identified QTLs with 8.1–21.2% phenotypic variance were mapped to five LGs. The regions of identified QTLs ranged from 51 to 527 kb, with an average length of 168 kb.

**Figure 2:**
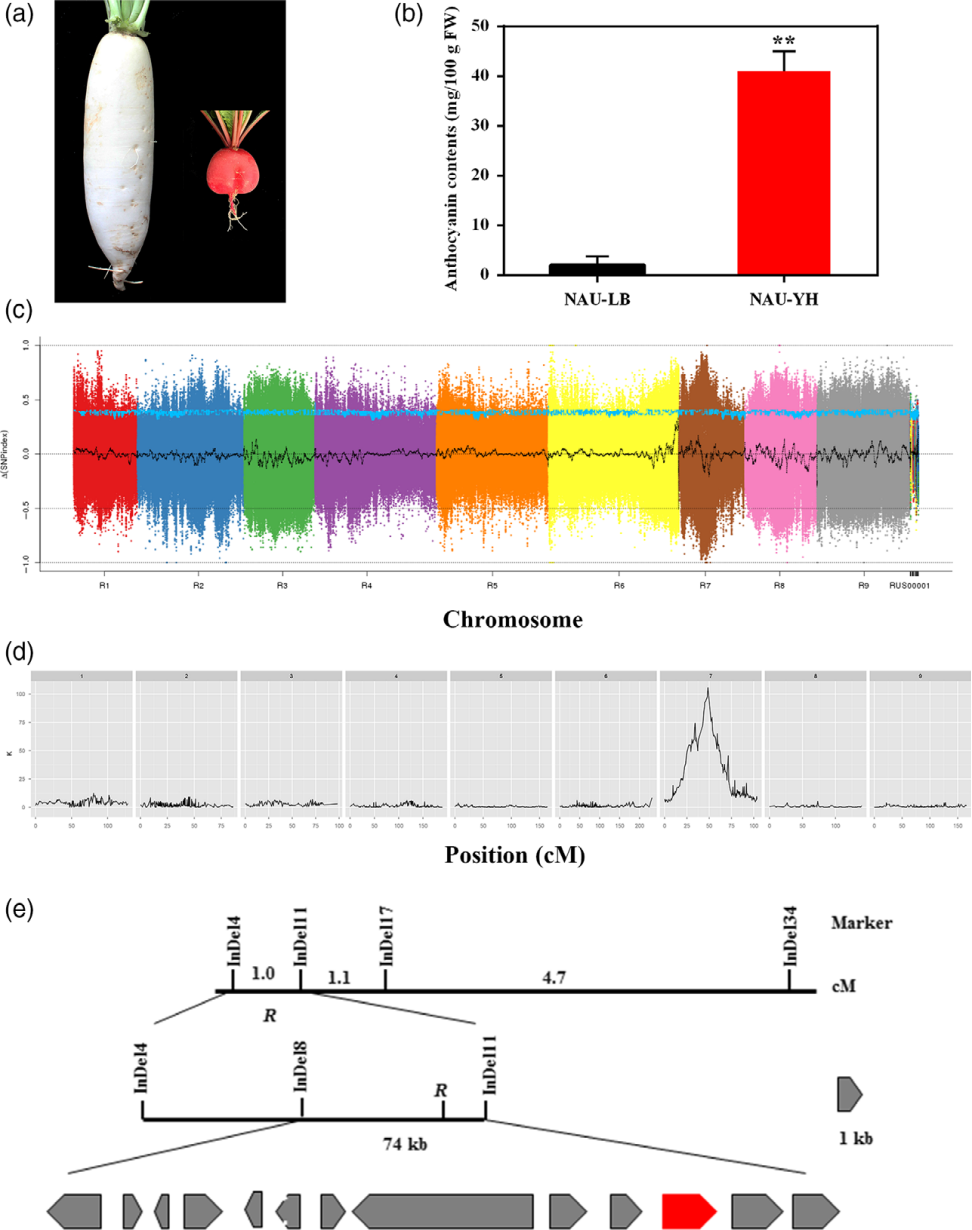
Map‐based cloning of red skin gene *R*. (a) Root skin colour of ‘NAU‐LB’ and ‘NAU‐YH’. (b) Anthocyanin contents in skin of ‘NAU‐LB’ and ‘NAU‐YH’. Bars represent means ± SD of three independent experiments. Asterisks indicate significant differences between ‘NAU‐LB’ and ‘NAU‐YH’ as determined by Student's *t* test (**, *P *<* *0.01) (c) Δ(SNP index) graph identified an interval of 9.0–11.6 Mb on chromosome 7 by BSA‐seq analysis. (d) QTL analysis for red skin colour using a Kruskal–Wallis test (K*) and 2852 bin markers. (e) The *R* locus was mapped on chromosome 7 between the markers RsInDel4 and RsInDel11 and was narrowed to a 74‐kb interval that included 13 ORFs. *Rs388430* is shown as the red box.

To isolate the taproot skin colour gene, the ‘NAU‐LB’ (white skin colour) was crossed with ‘NAU‐YH’ (red skin colour) (Figure 2a). The anthocyanin content of ‘NAU‐YH’ was higher than ‘NAU‐LB’ (Figure 2b). All F_1_ plants exhibited red root skin, and the F_2_ individuals were classified as red and white. The segregation of F_2_ generation fitted a 3:1 ratio in two consecutive years (χ^2^ = 0.002 in 2016 and χ^2^ = 0.896 in 2017). Trait segregation in BC_1_P_1_ populations was the ratio of 1:1 (χ^2^ = 0.06; Table 2). The taproot skin of all the BC_1_P_2_ populations was red. This genetic analysis confirmed that the root skin colour is controlled by a dominant gene, which was designated as *R*. To identify the causal gene underlying the taproot skin colour, clean reads from 10 red skin plants and 10 white skin plants were collected. A total of 187 million and 191 million clean reads were generated for the R‐bulk (47.26‐fold genome coverage) and W‐bulk (48.3‐fold genome coverage), respectively (Table S7). After eliminating the low‐quality SNP index in two bulks, 2 500 223 SNPs were identified on all nine chromosomes. A Δ(SNP index) graph was calculated and plotted against the genome positions, and the *R* locus was located to 2.6‐Mb (9.0–11.6 Mb) interval on chromosome 7 (Figure 2). A total of 719 938 InDels were identified between R‐bulk and W‐bulk, 34 of the InDels in candidate interval were selected to amplify polymorphisms among the parental lines and two DNA bulks. Ten polymorphic markers were used to screen the F_2_ individuals, and the *R* locus was anchored between the InDels markers RsInDel4 and RsInDel11. The *R* gene was narrowed down to a 74‐kb genomic region between the InDels markers RsInDel8 and RsInDel11 using 145 F_2_ plants with the white skin phenotype (Figure 2e).

**Table 2:** Inheritance of root skin colour in the mapping population

Population	Total plants	Red plants	White plants	Expected ratio	χ^2^	*P*
F_2_(2016)	137	103	34	3:1	0.002	0.961
F_2_(2017)	430	314	116	3:1	0.896	0.344
BC_1_P_1_(2017)	151	77	74	1:1	0.06	0.81
BC_1_P_2_(2017)	176	176	0	1:0	–	–

Chi‐square test for goodness of fit at 0.05 significance level (χ^2^_0.05:1_ = 3.84).

Quantitative trait locus analysis for taproot skin colour was performed using MapQTL based on the high‐density genetic map. A single locus (bin marker 2194) located on LG 7 was the most significant markers according to the Kruskal–Wallis test (*K *=* *105.83, *P *<* *0.0001; Figure 2d). The peak LOD in LG 7 was also located at bin 2194, with highest LOD values of 45.24 by interval mapping (Figure S4). Finally, the *R* locus was mapped to a 72‐kb region, indicating the high accuracy of high‐density genetic map in this study. Thirteen putative open reading frames (ORFs) were predicted based on the radish reference genome information (Table S8). An *Rs388430* was predicted to belong to the R2R3 MYB transcription factor family, which was a homologous of *AtMYB90*/*PAP2* involved in regulation of the anthocyanin biosynthetic pathway (Gonzalez *et al*., 2008). RT‐PCR and RT‐qPCR analysis revealed that the expression level of *RsMYB90* was higher in taproot skin of ‘NAU‐YH’ than that in ‘NAU‐LB’ (Figure S5a,b), indicating *RsMYB90* was the candidate gene for the *R* controlling the red skin colour of taproot. A total of 16 radish genotypes with different skin colours were employed to investigate the expression profiles of *RsMYB90* gene by RT‐PCR approach. As shown in Figure S5c, the *RsMYB90* gene is highly expressed in eight red skin varieties, whereas it is barely expressed in eight white skin varieties. Phylogenetic analysis of the deduced amino acid sequences showed that *RsMYB90* was most closely related to *Arabidopsis PAP2*,* MYB90*,* MYB113* and clustered with the several other anthocyanin‐related MYB TFs (Figure S6). The alignment of the amino acid sequences revealed that the R2R3 motifs were conserved at the N‐terminus and the *RsMYB90* has predicted interaction domains for bHLH partners, and a C‐terminal consensus sequence, indicating its potential roles in activation of anthocyanin biosynthesis (Figure S7).

### Comparison of the linkage map with the radish genome

**Figure 3:**
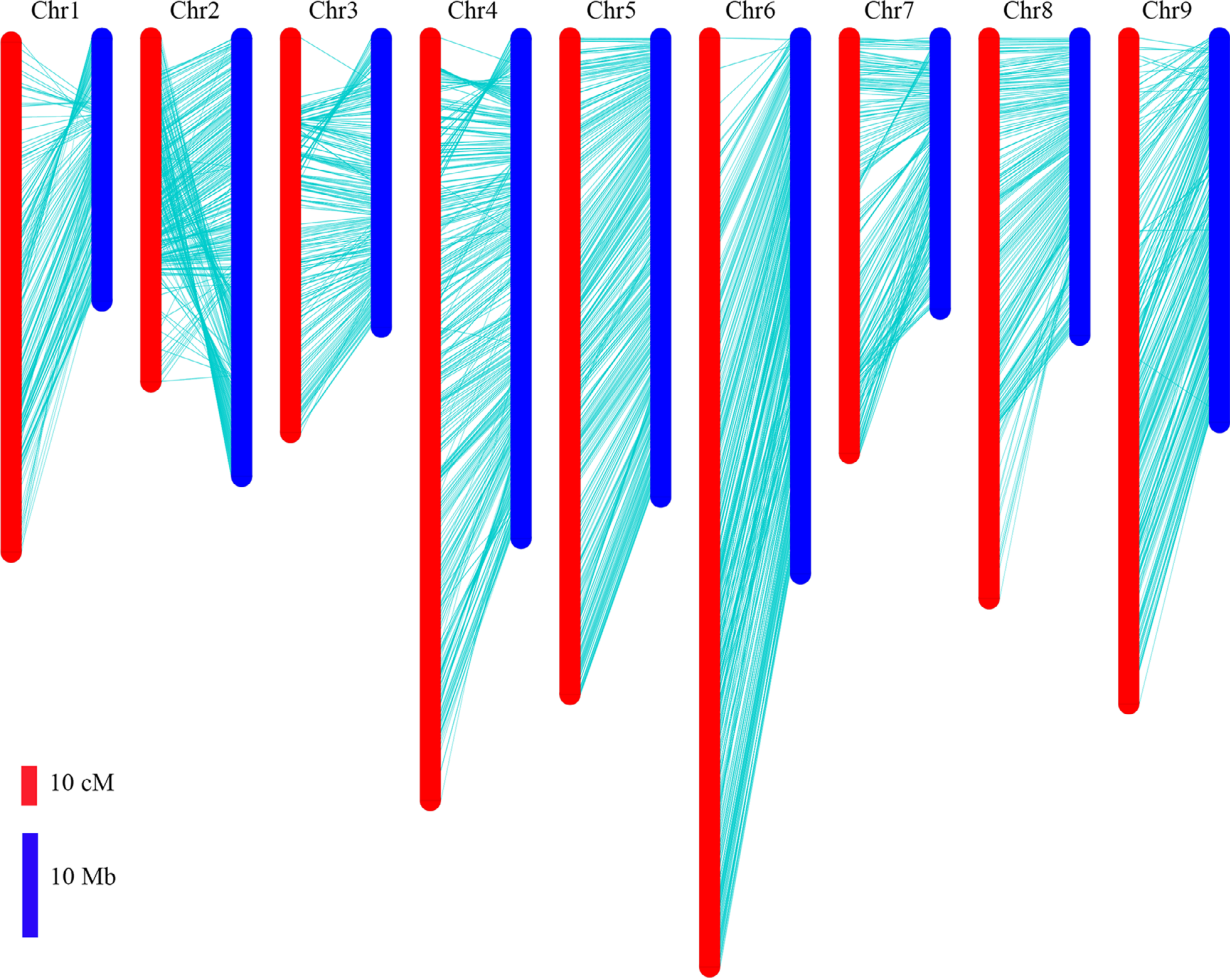
Comparison between the genetic map and radish genome sequence.

The locations of bin markers on the genetic map were compared with their physical positions in the radish genome assembly. All the bin markers were mapped on the nine pseudo‐chromosomes, representing 80.7% of 424 Mb for the radish reference genome (Figure 3). A high degree of collinearity was observed between the genetic map and the corresponding chromosome. However, there were still few regions displayed inconsistence on several chromosomes. The bins located at 26–43 Mb on chromosome 2 were inverted in upper region of the corresponding LGs. The order of bins on the distal ends of the chromosomes 1, 3 and 4 was inconsistent with the genetic map.

### Colinearity of the radish genetic map with *Brassica* genome

**Figure 4:**
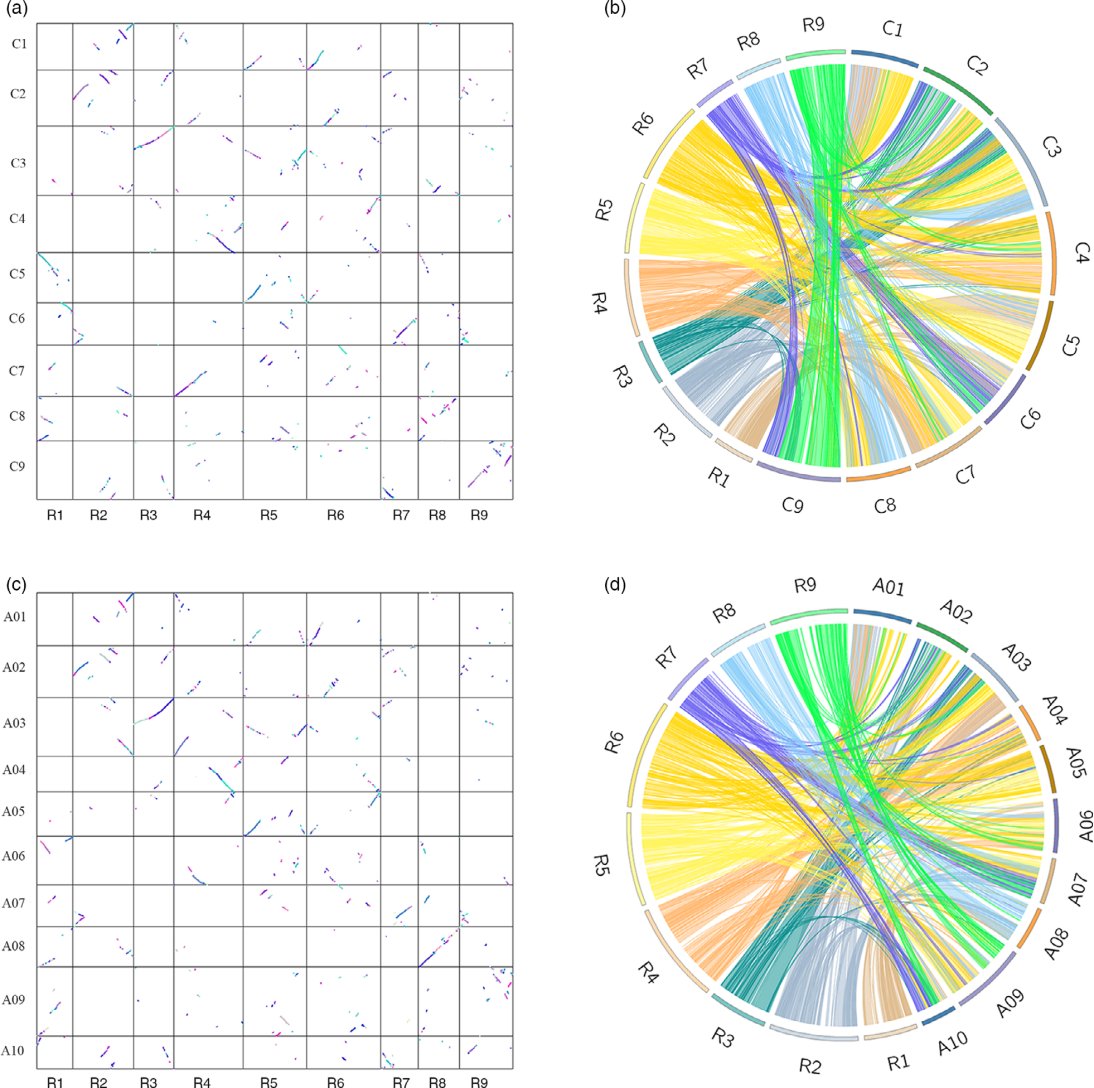
Pairwise dot‐plot comparison of the syntenic relationships between linkage map and *Brassica oleracea* (a) and *B. rapa* (c) pseudomolecules. Circos representation of the genome synteny between radish and *B. oleracea* (b) and *B. rapa* (d).

To gain insight into the evolutionary history of the radish genome, the location of bin markers was searched against the physical map of *B. rapa* and *B. oleracea* genomes. Among the 2852 bin markers, 2411 (84.5%) and 2385 (83.6%) markers were aligned to *B. rapa* and *B. oleracea* chromosomes, respectively. Synteny analysis revealed that the radish LG 8 and LG 9 showed higher conservation with *B. rapa* chromosome 8 and *B. oleracea* chromosome 9, respectively (Figure 4). In contrast, no one‐to‐one chromosome relationship was presented between the remaining radish linkage groups and the two *Brassica* species. Despite the radish shared the same chromosome number with *B. oleracea*, LG 7 of radish was matched to chromosomes 2, 3, 6 and 9 of *B. oleracea*, indicating that chromosomal rearrangements have occurred between radish and the two *Brassica* genomes after their divergence from the common ancestor.

### Recombination landscape in radish

**Figure 5:**
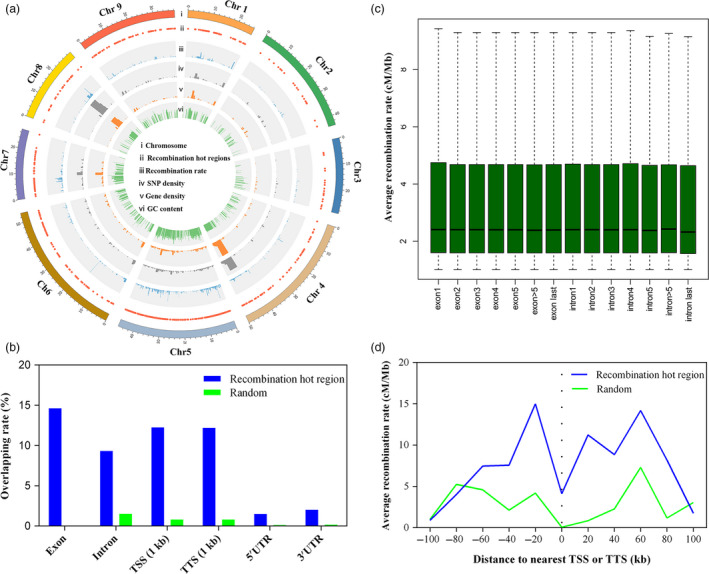
The fine‐scale profile of recombination hot regions (RHRs) around the genome. (a) Genomic features of RHRs: Genetic positions of the RHRs in the nine radish pseudo‐chromosome (ii), count of recombination rates (iii), SNP density (iv), gene density (v) and GC content (vi) within RHRs. (b) Overlap analysis of RHRs with various genomic features (blue) and random regions (green). (c) Recombination rate within exons and introns for genes. (d) Average recombination rate as a function of distance to nearest transcription start site (TSS) and transcription start site (TSS) for all genes in RHRs (blue) and random regions (green).

To provide a comprehensive overview of recombination in radish, the recombination rate along each chromosome was estimated by comparing genetic and physical distance. The average recombination rate across the entire genome was 3.8 cM/Mb. The high recombination in telomere regions of all nine chromosomes was observed, whereas the recombination was suppressed in the centromere regions (Figures 5 and S8). The number of recombination per chromosome was not significantly positively correlated with the physical length of the chromosomes (*r *=* *−0.02, *P *=* *0.9573; Figure S9). To detect recombination hotspots in the genome, the chromosomal regions with recombination rate greater than 1.0 cM/Mb were defined as recombination hot regions (RHRs). A total of 504 RHRs were identified and unevenly distributed on the nine LGs (Table S9). The length of RHR ranged from 6.1 to 850 kb with one hotspot detected on average every 215 kb. Chromosome 5 had the maximun RHRs, while chromosomes 2 and 3 had the fewest. The highest recombination rate in RHRs (39.97 cM/Mb) was observed on the chromosome 6 (Chr 6: 17 049 937–17 063 645), which was 10.5‐fold higher than the genome average recombination rate. Previous studies indicated that recombination hotspot was associated with GC content, nucleotide diversity and gene density (Silva‐Junior and Grattapaglia, 2015). The RHRs with high recombination rates were positively correlated with SNP density, gene density and GC content, respectively, which was predominately located in chromosome ends (Figure 5a). Correlations between GC content and recombination rate are consistent with the result that the increased recombination rates drive increasing GC content by the process of GC‐biased gene conversion (gBGC) (Singhal *et al*., 2015).

To investigate the localization of RHRs in the genome, the overlap between RHRs and exons, introns, 5′ UTRs, 3′ UTRs, 1 kb upstream of TSS (transcription start sites) and 1 kb downstream of TTS (transcription termination sites) was tested. In comparison with random regions, a higher proportion of overlap with exons, introns, 5′ UTRs, 3′ UTRs, 1 kb TSS and 1 kb TTS was observed in RHRs. The RHRs overlapped exon (14.6%) were higher than introns (9.3%), and recombination rates were higher in first exon than in last intron for genes that have ≥5 exons (Figure 5b,c). A higher proportion of overlap was enriched near 1 kb TSS (12.3%) and 1 kb (12.2%) TTS, which could be presumed that TSSs and TTSs facilitated RNA polymerase II (Pol II) transcriptional regulation (Choi *et al*., 2013). Assessments on the average fine‐scale recombination rate profiles relative to all genes indicated that the average recombination rate was increased near TSSs and TTSs, and decreased within the transcribed region, in agreement with the random regions (Figure 5d). Gene Ontogeny (GO) analysis showed that the predominant GO terms enriched in RHRs were related to metabolic process and organic substance metabolic process in biological processes (Figure S10). In the cellular component category, binding and catalytic activity represented the most abundant significantly enriched subcategory, demonstrating genes within RHRs may play an important role in metabolic process.

To explore the recombination under genetic control, the detected QTLs were searched against the RHRs. As expected, three QTLs for three traits were located in RHRs. A major QTL originated from recombination (*qRL8*) with 8.3% of total phenotypic variance was mapped to bin 2355 of chromosome 8, with a physical length of 114 kb. Twenty‐three genes were identified in candidate region, one of which (*Rs407980*) encoding a sugar transporter 2 was homologous with *AtSTP2*. Previously studies showed that sugar transport proteins played important roles in sugar acquisition by roots in *Arabidopsis* (Chen *et al*., 2015). It was indicated that the *Rs407980* was involved in sugar transport system and acted a candidate gene for root length in radish. Taken together, it could be infered that the recombination plays vital role in phenotypic variation of radish.

## Discussion

A high‐density genetic map can be efficiently used to identify candidate causal genes for important agronomic traits and comparative genomics, and support the assembly of reference genome. Radish is an important root vegetable crop worldwide, and the insufficiency of molecular markers hindered the effective application of linkage map for MAS of horticulture traits in breeding programmes. In this study, a high‐density bin map composed of 2852 bin markers was constructed and applied to fine mapping of a candidate gene for red skin colour. Comparative genomic analysis provided insights into the evolutionary history of the radish and *Brassica* genome. This study represents a first report on the comprehensive characterization of recombination landscape in radish. These results provided valuable information on the understanding of genome features and the practice of molecular breeding in radish.

### High‐density linkage map for breeding programme and genome assembly

The sufficiency of molecular markers, population size and the highly efficient genotyping approaches could facilitate improving the resolution of QTL mapping and ultimately map‐based cloning. The WGRS and subsequent analyses provide an effective way to develop a high density genetic map (Gao *et al*., 2013; Hu *et al*., 2017). In this study, a high‐density genetic map with 2852 recombinant bins was constructed and covered 1306.8 cM with an average marker interval of 0.46 cM. Previously, an integrated linkage map using 221 SNPs and 1514 traditional molecular markers was constructed (Kitashiba *et al*., 2014). Mun *et al*. (2015) reported a genetic map with 2532 SNPs and 146 PCR‐based markers developed from resequencing. However, the marker density of these maps is still limited to pinpoint the genes underlying a considerable number of QTLs. The marker density in the present linkage map is significantly higher than that of many reported radish maps (Kitashiba *et al.*, 2014; Mun *et al.*, 2015; Xu *et al.*, 2012). A total of 18 QTLs controlling 11 traits were identified with this high‐density map, and the QTLs for the content of soluble solid, soluble sugar and soluble protein were firstly reported in radish. The QTL intervals detected in this study were significantly shorter than previously reported, indicating the improved resolution of the linkage map (Yu *et al.*, 2016).

The red skin with high concentration of anthocyanin is an important agronomic trait in modern radish breeding, but the underlying molecular bases have not been fully dissected. High‐density genetic map and whole‐genome resequencing‐based BSA are two effective methods to identify genetic loci associated with a specific trait (Takagi, 2013; Xu *et al.*, 2013). A *PyMYB114* gene on LG3 associated with red skin was identified using a high‐density genetic map in pear (Yao *et al.*, 2017). The *virescent‐1* gene has been rapidly and reliably characterized using BSA combined with whole‐genome sequencing in cotton (Zhu *et al.*, 2017). Here, the *RsMYB9*0 gene belonging to the R2R3 family was identified by combining BSA with whole genomic resequencing and map‐based cloning. QTL mapping for red skin further confirmed the reliability of the high‐density bin map in this study. It is known that R2R3 MYB transcription factors play essential roles in regulation of anthocyanin biosynthesis in many plants. In apple, *MYB1* and *MYBA* genes mapped to the same locus are responsible for skin colour (Ban *et al.*, 2007; Takos *et al.*, 2006). Previous studies have suggested that three *PavMYB10.1* alleles controlled the fruit skin colour in sweet cherry (Jin *et al.*, 2016). Phylogenetic analysis showed that the *RsMYB90* gene was highly homologous with *AtPAP2*. Previous studies revealed that the *MYB90/PAP2* gene was involved in anthocyanin accumulation in several plant species such as *Arabidopsis* and *Brassica napus* (Ilk *et al.*, 2015; Fu *et al.*, 2017). In *Arabidopsis*, the gene expression of *AtMYB90/PAP2* was down‐regulated with obvious anthocyanin deficiencies by RNAi (Gonzalez *et al.*, 2008). Overexpression of *OvPAP2* gene in *B. napus* produced red anthers and petals, illustrating the feasibility of producing red‐flowered oilseed rape (Fu *et al.*, 2017). In this study, co‐segregation of *RsMYB90* transcript with the skin colour indicated the *RsMYB90* gene might be responsible for the skin colour trait, and play an important role in anthocyanin accumulation in radish. Sequence analysis indicated that three SNPs were found in the coding region of *RsMYB90* gene between the P_1_ (white skin radish)and P_2_ (red skin radish) line, which resulted in three amino acid changes at the position of 55, 96 and 216 amino acids in proteins between parental lines (Figure S11a). Furthermore, a total of three InDels and SNPs were identified in the promoter regions of the two parental lines (Figure S11b). Previous studies indicated that the InDel and SNP markers in the promoter sequences play important roles in plant phenotype determination (Ye *et al.*, 2017; Zhang *et al.*, 2018). It could be speculated that these InDels and SNPs in the coding and promoter regions of two parental lines might lead into differential expression of *RsMYB90* gene and affect anthocyanin biosynthesis. Undoubtedly, further functional characterization of these InDels and SNPs would contribute to clarify the molecular regulatory mechanism underlying anthocyanin synthesis in radish. The MYB‐bHLH‐WD40 (MBW) complexes that regulated the genes involved in the late steps of anthocyanin and proanthocyanidin biosynthesis, which have been characterized in many plant species, including *A. thaliana* (Gonzalez *et al.*, 2008), apple (Xie *et al.*, 2012), maize (Kong *et al.*, 2012) and pear (Yao *et al*., 2017). In this study, the *RsMYB90* has conserved domain to interact with *bHLH*, indicating that *RsMYB90* plays a protective role in control of root skin anthocyanin biosynthesis in radish. These results showed that our high‐density genetic map was effective for genetic mapping of important agronomic traits and available to marker‐assisted selection in radish breeding.

Alignment of present genetic map to the radish ‘WK10039’ genome revealed a remarkable collinearity, indicating the robust assembly of reference genome. The relative order of markers in some regions showed nonuniformity between the LGs and the corresponding chromosomes, indicating the intrachromosomal rearrangements and genome reshuffling events have occurred between the two lineages (Du *et al.*, 2016). High‐quality genetic map played a vital role in anchoring scaffold sequences into pseudo‐chromosomes (Li *et al.*, 2017). In radish, 116 Mb (21.8%) of the cv. ‘Aokubi’ scaffolds was anchored to linkage group (Kitashiba *et al.*, 2014), and the other two genomes lacking genetic maps are not anchored into pseudo‐chromosomes (Mitsui *et al.*, 2015; Moghe *et al.*, 2014). In the present study, the bin markers in genetic map captured 344 Mb (80.7%) of the ‘WK10039’ genome sequence. Therefore, the high‐density linkage map constructed in this study would be useful for QTL mapping and provide a valuable resource to further improve the genome assembly in radish.

### Comparative analysis of the homoeologous relationships between radish and two *Brassica* genomes

The high‐density genetic map provided an opportunity to perform a comparison of collinearity between closely related species. Phylogenetic analysis revealed that the *Raphanus* was phylogenetically closest to *B. oleracea* and *B. rapa* (Jeong *et al*., 2016). Both *Raphanus* and *Brassica* belong to the Brassicaceae family, whose divergence was estimated at 7‐14 million years ago (Mya) after recent whole‐genome triplication (WGT) event in a common hexaploid ancestor (Cheng *et al.*, 2017; Jeong *et al*., 2016). It has been suggested that the basal chromosome number of nine in radish was derived from the diploid ancestor with seven chromosomes, and the proto‐*Raphanus* karyotype with 11 chromosomes underwent WGT event (Cheng *et al.*, 2017). In this study, a relatively weak syntenic relationship between radish and the two *Brassica* genomes was observed, despite the radish and *B. oleracea* have a common 9 haploid chromosome. The complex patterns of conserved synteny were accordant well with previous finding that the *Brassica* chromosomes have occurred extensive chromosomal breakages and fusions, which was probably a result of mesopolyploidy genome in *Brassica* (Liu *et al*., 2014). Previous studies demonstrated that the chromosome 1 and chromosome 2 between *B. rapa* and *B. oleracea* displayed high level of collinearity by comparative genomic analyses (Chalhoub *et al*., 2014). In this study, radish LG1 and LG2 were matched to three chromosomes of *B. rapa* and *B. oleracea*, illustrating the more chromosomal rearrangements resulted in a faster rate of genome evolution in radish. Taken together, our results provide insights into the comparative and evolutionary genomics research between the radish and the two Brassica species.

### Recombination contributed to phenotypic variation and breeding applications

Meiotic recombination is a fundamental biological process that generates genetic variation and new allele combinations, which have a critical role in phenotypic variations and genome evolution (Si *et al*., 2015; Singhal *et al*., 2015). The high‐resolution genetic map reported in this study provides a genome‐wide recombination ratio of 3.8 cM/Mb, which is in the similar range as can be calculated from *Arabidopsis* (Drouaud *et al*., 2006) and rice (Wu *et al*., 2003), but is higher than earlier estimates from cotton (1.8 cM/Mb) (Wang *et al*., 2015) and maize (0.9 cM/Mb) (Pan *et al*., 2016). Recombination varied across all the chromosomes and displayed a low recombination at telomeres, which are known to be highly heterochromatic (Wang *et al*., 2015). Some studies have reported that a higher recombination rate was observed in domesticated plants species as compared with their wild progenitors, illustrating that the high recombination rates are an adaptation favoured under artificial selection (Ritz *et al*., 2017; Si *et al*., 2015). The 504 RHRs identified in radish genome may help to facilitate marker‐assisted selection and shorten the breeding period by construction of populations with a higher recombination rate in specific genome regions.

Previous studies have showed that recombination rates are increased near annotated TSSs and TTSs and decreased within the transcribed region in animals (Auton *et al*., 2012; Singhal *et al*., 2015) and plants such as *A. thaliana* (Choi *et al*., 2013). In the present study, the increase in recombination rates near TSSs and TTSs supports the viewpoint mechanism that the recombination occurs at TSSs and TSSs is very close to the recombination machinery (Singhal *et al*., 2015). The density of disease resistance genes showed a significant positive correlation with the recombination rates in the rice genome (Si *et al*., 2015). The intergenic recombination has a potential effect on phenotypic variation in maize (Pan *et al*., 2016). These findings indicated that the frequency and distribution of recombination plays an important role in influencing the progress of breeding programme.

The high recombination rate was observed in rapidly environment changes (Ritz *et al*., 2017). In the current study, the genes within RHRs showed the significantly enriched GO terms associated with regulation of metabolic process and organic substance metabolic process in biological processes. These results were consistent with the observation that plant metabolism contributes to face unavoidable environmental changes (Silva‐Junior and Grattapaglia, 2015). In sharp contrast to rice, frequent recombination primarily involved in response to environmental stimuli (Si *et al*., 2015). Accordingly, adaptation in a variable environment with different strategies might exist in plants and a maximize recombination rate would be adopted for populations under selection. Many studies showed that the regulatory region containing recombination is significantly related to quantitative variation in expression level and generated adaptive genetic variations during crop domestication and improvement (Pan *et al*., 2016; Si *et al*., 2015). Also, the intragenic recombination had potential effects on gene expression and phenotypic variation in maize (Pan *et al*., 2016). It was found that recombination events were occurred in three QTLs, and *Rs407980* for root length involved in metabolic process was associated with recombination hotspot. These results could provide fundamental insight into complex landscape of recombination in radish and act as a reference to investigate recombination rate variation in Brassicaceae.

In conclusion, a high‐density genetic map with high resolution and accuracy was constructed through whole‐genome sequencing. A candidate causal gene for taproot red skin was identified using a combination of genome‐based QTL mapping and map‐based cloning. *RsMYB90* gene plays an important role in regulating anthocyanin biosynthesis, and the identification of *RsMYB90* is useful for further genetic manipulation of anthocyanin accumulation in radish. The high‐density genetic map was applied for comparative genomic study and the exploration of recombination landscape at whole‐genome scale in radish, which would provide a robust basis for further investigation on genetic mapping and genomic recombination and facilitate further molecular breeding in radish.

## Experimental procedures

### Plant materials and phenotype data collection

Two advanced radish inbred lines, ‘NAU‐LB’ with white taproot skin and ‘NAU‐YH’ with the red taproot skin, were used in this study. The ‘NAU‐LB’ (P_1_) was crossed with ‘NAU‐YH’ (P_2_) to produce the F_1_, F_2,_ BC_1_P_1_ and BC_1_P_2_ populations. The F_2_ populations consisted of 137 individuals were used for linkage analysis and genetic map construction. A F_2_ population (430 lines) derived from the same cross was developed for fine mapping of genes associated with root skin colour. All the plants were grown in the JiangPu Breeding Station of Nanjing Agricultural University. To dissect the genetic mechanism underlying important horticulture traits in radish, the phenotypic data of ten traits, including root length, root weight and plant height, were measured from the F_2_ individuals and their two parental lines. The pigment phenotypes of root skin were investigated visually. For genetic analysis, chi‐square tests (χ^2^) were conducted to evaluate the goodness of fit of segregation ratios in F_2,_ BC_1_P_1_ and BC_1_P_2_ populations.

### Population resequencing and genotyping

The genomic DNA of the two parents and F_2_ plants was extracted from the fresh leaf tissue following the CTAB (cetyl trimethylammonium bromide) method with minor modifications (Liu *et al.*, 2003). The libraries were constructed with an insert size of 180 bp and sequenced on the Illumina HiSeq 2500 platform according to the manufacturer's standard protocols. Reads with ≥10% unidentified nucleotides and >50% bases with Phred quality <5 were filtered before alignment. The sequencing reads were aligned to the radish reference genome (‘WK10039’ http://radish-genome.org/) using Burrows–Wheeler Aligner (BWA) tools with the parameters of ‘mem ‐t 4 ‐k 32 ‐M ‐R’ (Li and Durbin, 2009). Alignment files were converted into BAM files using the sort setting in SAMtools software (Li *et al.*, 2009). Only the reads mapped uniquely to the reference genome sequence were then used to call SNPs. Identification of SNPs between the parental lines and F_2_ individuals was performed with the GATK software (McKenna *et al.*, 2010). Polymorphic markers between the two parental lines with aa × bb segregation pattern were retained and genotyped for F_2_ individuals. High‐quality SNPs were obtained with a custom Perl script by the following criteria: the minimum sequencing depth of each SNP allele was 4, and an allele represented at least 30% F_2_ plants with SNPs.

### Phenotype evaluation

The root skin colour was defined as red or white. The leaf number (LN) was measured by artificial statistics. Plant height (PH), leaf width (LW), root length (RL) and root diameter (RD) were measured using Vernier calipers. Plant weight (PW) and root weight (RW) were measured using electronic scales. The content of total soluble solid content (TSS) was measured using a refractometer (Atago, model N‐1). The content of soluble sugar (SS) and soluble protein (SP) was measured as described by Su *et al.* (2016).

### Determination of total anthocyanin content

Total anthocyanins were extracted from 0.3 g radish taproot skin using the pH differential method described by Jiang *et al.* (2016). The absorbance of the solutions was measured by a multimode microplate reader (Infinite M200, Tecan Inc., Mannedorf, Switzerland). The anthocyanin content was calculated with the following formula: TA = *A* ×MW × 5 × 100 × *V*/ε, where TA is the total anthocyanin content (mg/100 g fresh weight), *A = *[A_510 nm_ (pH 1.0) − A_700 nm_ (pH1.0)] − [A_510 nm_ (pH 4.5) − A_700 nm_ (pH 4.5)], MW stands for molecular weight of cyanidin‐3‐glucoside (MW = 449.2), *V* stands for final volume (mL), and ε is the extinction coefficient of cyanidin‐3‐glucoside (ε = 26 900). The analyses of each sample were performed in triplicate.

### Bin map construction

Chi‐square test was conducted to detect the ratio of marker segregation, and markers with significantly distorted segregation (*P*‐value < 0.001) were filtered for further analysis. A slightly modified sliding window approach was adopted to calculate the ratio of SNP alleles derived from ‘NAU‐LB’ and ‘NAU‐YH’ and identified recombinant breakpoints (Huang *et al*., 2009). A window size of 15 SNPs with no missing was used for genotyping calling. Windows with 11 or more SNPs from either parent were considered to be homozygous for an individual, while those with less were called as heterozygous. Consecutive 100‐kb intervals with the same genotype in the entire F_2_ population were clustered as a recombination bin. Bins served as genetic markers were employed for the construction of the genetic linkage map using JoinMap 4.0 with a recombination frequency <0.4 and minimum logarithm of odds (LOD) scores of 6 (Van Ooijen, 2006). The relationships between the genetic and physical positions were visualized using ALLMAPS program (Hu *et al*., 2017).

All the bin makers were tested for significant segregation distortion using a chi‐square test. The log_10_ value of the chi‐square test statistic for each marker was plotted against marker position using the R package qqman. A region containing three or more closely linked bins exhibiting significant segregation distortion (*P *<* *0.005) was defined as the SDR.

### QTL mapping

Quantitative trait locus analysis for 11 traits was performed using the MAPQTL 6.0 software (Van Ooijen, 2009) followed the procedure described by Du *et al*. (2016). The nonparametric Kruskal–Wallis (KW) test was used to test the association between marker and traits at significance *P *≤* *0.001. Interval mapping method was conducted to detect the significance of QTLs associated with skin colour. The linkage groups (LGs) and the detected QTLs were drawn with MapChart version 2.2 (Voorrips, 2002).

A QTL‐seq method with minor modification was used to perform the QTL analysis in radish (Abe *et al*., 2012; Takagi, 2013). Two DNA bulks, red bulk (R‐bulk) and white bulk (W‐bulk), were constructed by collecting the clean reads of 10 red skin individuals and 10 white skin individuals, respectively. All reads obtained from two bulks were mapped on to the radish genome sequence with BWA software (Li and Durbin, 2009). SNP calling was performed using GATK software (McKenna *et al.*, 2010). SNP positions with SNP indices of both bulks less than 0.3 and read depth <7 were discarded. SNPs with SNP index missing in either bulk were also excluded. The SNP index and Δ (SNP index) were employed to identify candidate regions for skin colour gene (Takagi, 2013). The average distribution of Δ (SNP index) of the SNPs at a given genomic interval was computed using a sliding window analysis of 1 Mb window size and 10 kb increment. The SNP index graphs for R‐bulk and W‐bulks, as well as corresponding Δ (SNP index) graph, were plotted. The red skin gene was firstly mapped with InDel (Insertion/Deletion) markers using F_2_ individuals and further narrowed by 145 individuals with white skin from combined F_2_ population (Table S10). Linkage analysis was conducted using JoinMap 4.0 with an LOD threshold score of 6.0.

### Genome synteny and characterization of RHRs

MCScanX was employed to detect syntenic regions (Wang *et al*., 2012), and consensus sequences of all mapped SNP markers were searched against the genome sequences of *R. sativus*,* B. rapa* and *B. oleracea* using BLASTX with a cut‐off E‐value < 1.0E‐10. Marker with multiple hits was filtered, and only the best hits were employed. The graphical comparative maps were visualized using Circos program (http://circos.ca/).

The estimation of recombination rate (cM/Mb) was calculated using in‐house Perl scripts, which divided the genetic length of the segment in cM by the physical length of the segment in Mb. The bin marker interval with recombination rate greater than 1.0 cM/Mb was defined as RHRs. The relationships between recombination rate within RHRs and genomic features (SNP density, gene density and GC content) were tested. The overlapping rate of RHRs with exons, introns, 5′ UTRs, 3′ UTRs, 1 kb TSS and 1 kb TTS was measured. The randomly permuted genomic regions were assessed for their overlap with each feature. The genes overlapped RHRs were conducted to perform gene ontology (GO) category enrichment analysis.

### Quantitative real‐time PCR (RT‐qPCR) analysis

Total RNA was extracted from root skin of ‘NAU‐YH’ and ‘NAU‐LB’ using the TRIzol Reagent (Invitrogen, Carlsbad, CA, USA) kit. cDNA was synthesized using the SuperScript III First‐Strand Synthesis System (Invitrogen). The PCR and amplification were performed as described previously (Xu *et al*., 2015a). RT‐qPCR analysis was carried out with SYBR Green PCR Master Mix (TaKaRa) on a ROCHE LightCycler 480 instrument according to the manufacturer's instructions. Reactions were run with three biological replicates. The radish *Actin* gene was used as an internal control. The relative expression of each target gene was normalized with the 2^−ΔΔC^_T_ algorithm (Livak and Schmittgen, 2001). All the primer sequences are listed in Table S10.

## Conflict of interest

The authors declare no competing financial interests.

## Author’ contributions

X.L. performed the data analysis and wrote the manuscript. M.T. and J.D. conducted validation of experiments. L.F. and Y.W. contributed powerful analytical tools. Y. C and Y.Z. helped with the revise of the manuscript. L.L. and L.X. conceived and designed the research. All authors read and approved the final manuscript.

## Supporting information


**Figure S1** A high‐density linkage map with bin markers of radish.
**Figure S2** Distribution of distorted segregating bins across the radish genome.
**Figure S3** Frequency distribution of ten traits in F_2_ population of radish.
**Figure S4** Interval mapping analysis on the LG7 for red skin colour of radish taproot.
**Figure S5** Expression profiling of *RsMYB90* in taproot of two parental lines (a,b) and different genotypes (c).
**Figure S6** Phylogenetic tree of RsMYB90 and R2R3 MYBs of other plant species.
**Figure S7** Protein sequence alignment of RsMYB90 and R2R3 MYB proteins from different species.
**Figure S8** Genetic *versus* physical distance maps of radish nine chromosomes.
**Figure S9** The relationship between recombination rate and physical length of the radish chromosomes.
**Figure S10** Gene ontology enrichment of the genes within recombination hot regions (RHRs).
**Figure S11** Amino acid sequence (a) and promoter sequence (b) of *RsMYB90* gene in the two parental lines.

**Table S1** Statistics of sequencing data.
**Table S2** The distribution of bin markers in linkage group.
**Table S3** Segregation distortion markers in F_2_ population at a significance level of *P *<* *0.005.
**Table S4** The distribution of segregation distortion regions.
**Table S5** Correlation coefficients among ten traits in radish.
**Table S6** QTLs analysis for ten traits in radish.
**Table S7** The statistics analysis of the sequencing data in R‐bulk and W‐bulk.
**Table S8** Gene prediction for the candidate interval.
**Table S9** The location of recombination hot regions (RHRs) on the radish chromosome.
**Table S10** Primer sequences used in this study.
