# Supplementary material for: An ultra‐high‐density genetic map provides insights into genome synteny, recombination landscape and taproot skin colour in radish (Raphanus sativus L.)
Source: Plant Biotechnol J. 2019 Jul 4;18(1):274–86. doi: 10.1111/pbi.13195 (PMC6920339; doi:10.1111/pbi.13195)
Supplement: Supplementary file 1 — Figure S1 A high‐density linkage map with bin markers of radish. Figure S2 Distribution of distorted segregating bins across the radish genome. Figure S3 Frequency distribution of ten traits in F2 population of radish. Figure S4 Interval mapping analysis on the LG7 for red skin colour of radish taproot. Figure S5 Expression profiling of RsMYB90 in taproot of two parental lines (a,b) and different genotypes (c). Figure S6 Phylogenetic tree of RsMYB90 and R2R3 MYBs of other plant species. Figure S7 Protein sequence alignment of RsMYB90 and R2R3 MYB proteins from different species. Figure S8 Genetic versus physical distance maps of radish nine chromosomes. Figure S9 The relationship between recombination rate and physical length of the radish chromosomes. Figure S10 Gene ontology enrichment of the genes within recombination hot regions (RHRs). Figure S11 Amino acid sequence (a) and promoter sequence (b) of RsMYB90 gene in the two parental lines. [file PBI-18-274-s001.docx]

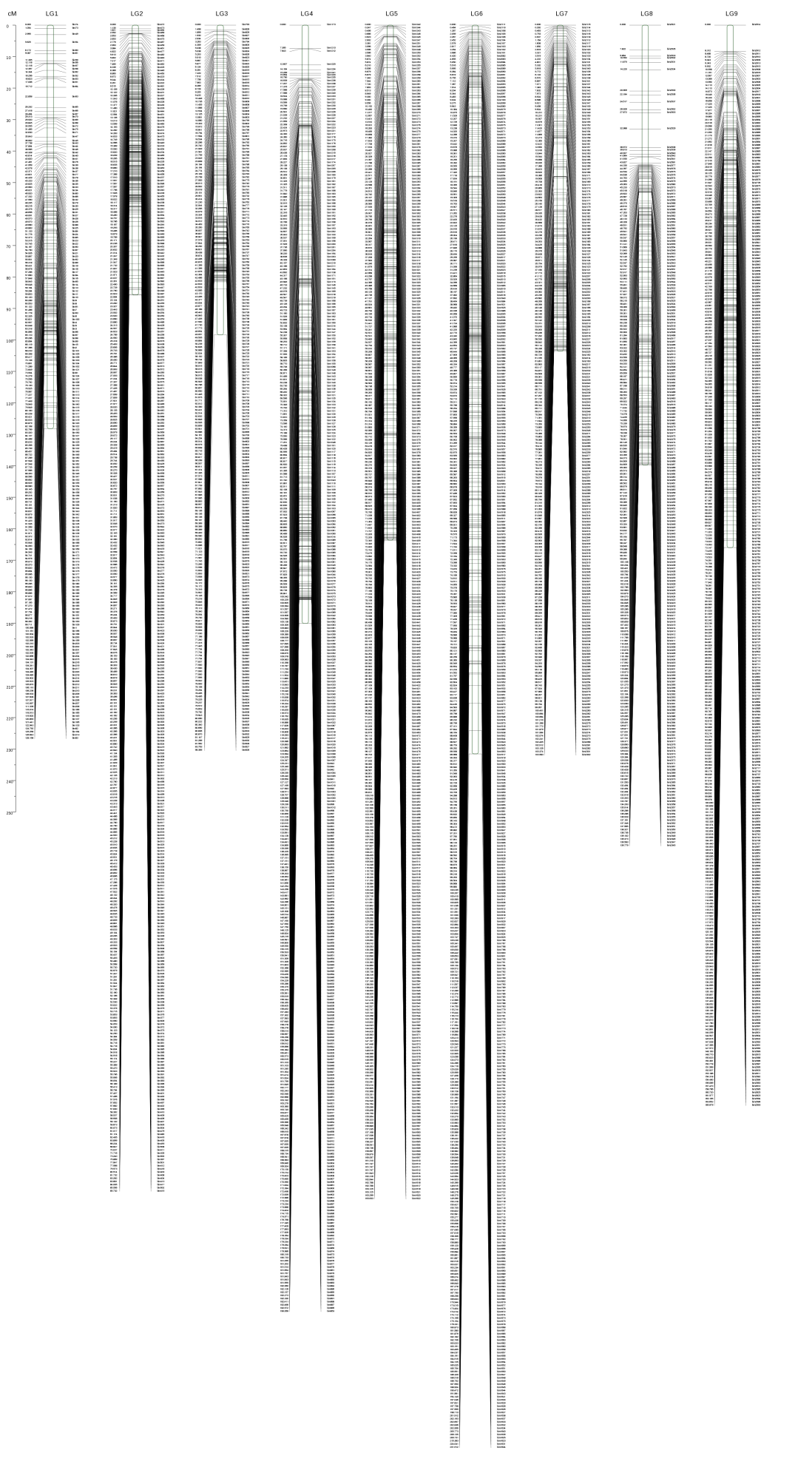


**Figure S1.** A high-density linkage map with bin markers of radish.

**
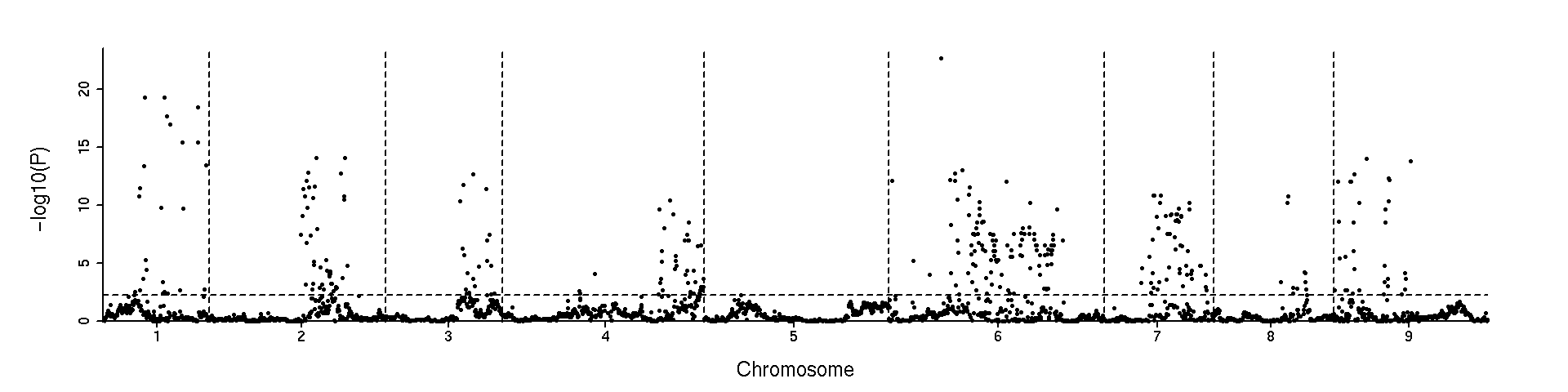
**

**Figure S2.** Distribution of distorted segregating bins across the radish genome. The guideline indicates the significance threshold of the chi-square test at *P* = 0.05.

**NAU-YH**

**NAU-LB**

**NAU-LB**

**NAU-YH**

**NAU-LB**

**NAU-YH**

**NAU-LB**

**NAU-YH**

**NAU-YH**

**NAU-YH**

**NAU-LB**

**NAU-LB**


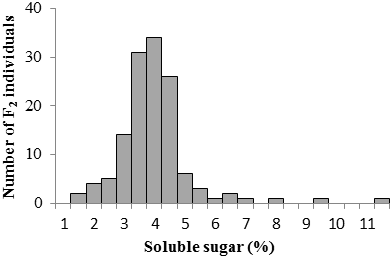

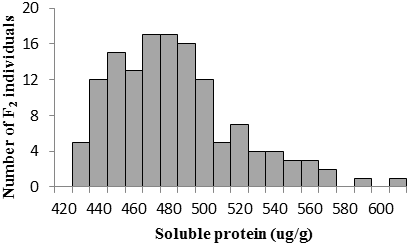

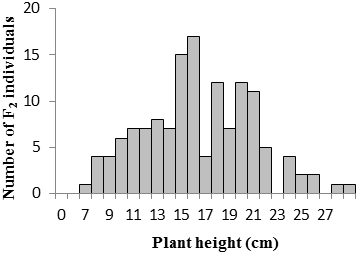

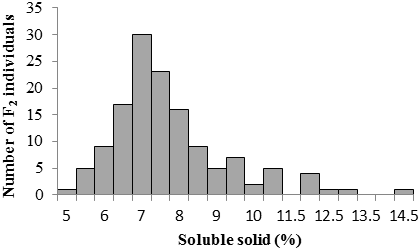

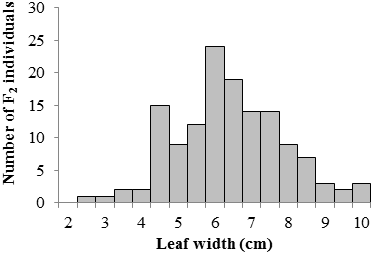

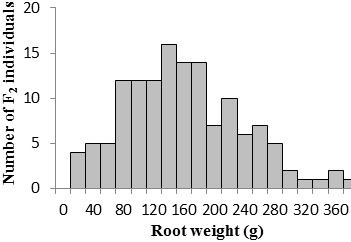

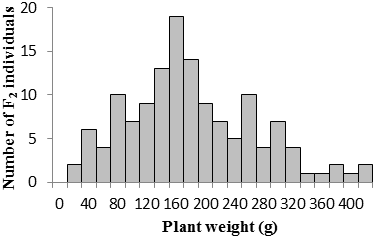

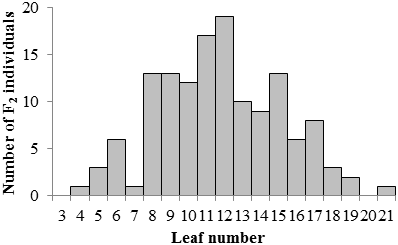

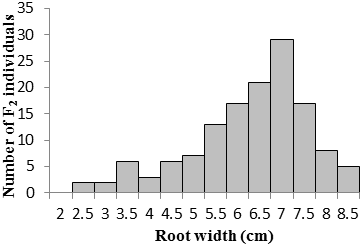

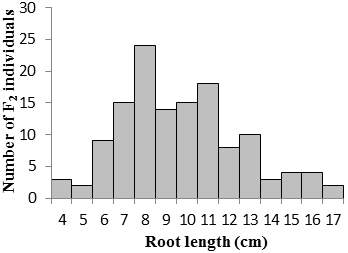


**Figure S3.** Frequency distribution of ten traits in F_2_ population of radish.

**NAU-YH**

**NAU-LB**

**NAU-YH**

**NAU-LB**

**NAU-YH**

**NAU-LB**

**NAU-LB**

**NAU-YH**

**
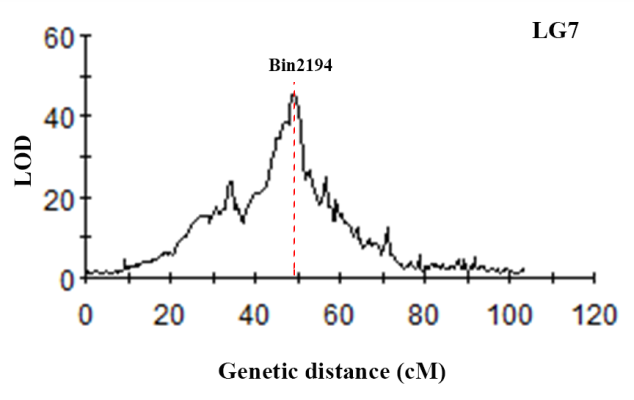
**

**Figure S4.** Interval mapping analysis on the LG7 for red skin color of radish taproot**.**


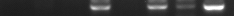


1 2 3 4 5 6 7 8


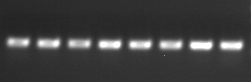


***RsMYB90***

***Actin***

(a)

(b)


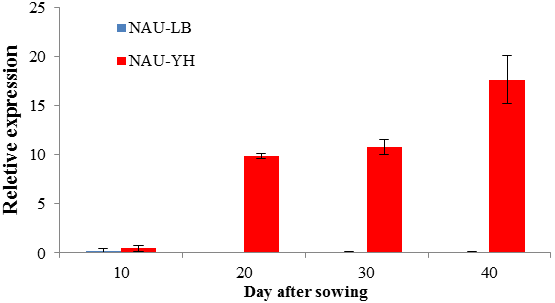


M 1 2 3 4 5 6 7 8 9 10 11 12 13 14 15 16


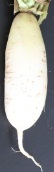

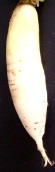

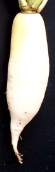

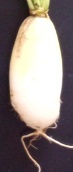

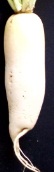

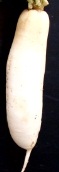

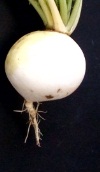

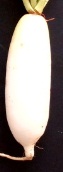

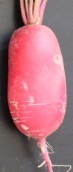

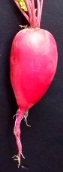

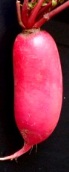

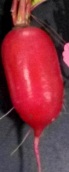

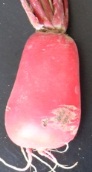

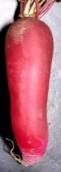

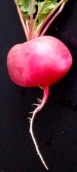

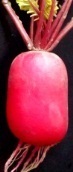


1 2 3 4 5 6 7 8 9 10 11 12 13 14 15 16


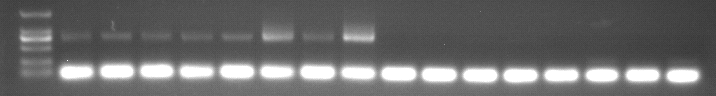

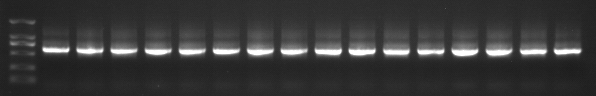


***RsMYB90***

***Actin***

**Radish**

(c)

**Figure S5.** Expression profiling of *RsMYB90* in taproot of two parental lines by RT-qPCR. (a) and RT-PCR (b). 1,3,5,7 represent the taproot of the ‘NAU-LB’ and 2,4,6,8 represent the taproot of ‘NAU-YH’ at different times, respectively, (c) Expression profiles of *RsMYB90* gene in different radish genotypes. Red skin genotypes (1-8)：‘HTG’, ‘HYU’, ‘MSG’, ‘THL’, ‘DHP’, ‘CH’, ‘YHA’, ‘TXH’; White skin genotypes (9-16): ‘XBH’, ‘SHY’, ‘XBY’, ‘XQB’, ‘WRZ’, ‘SN3’, ‘QSGG’, ‘NZL’.

**
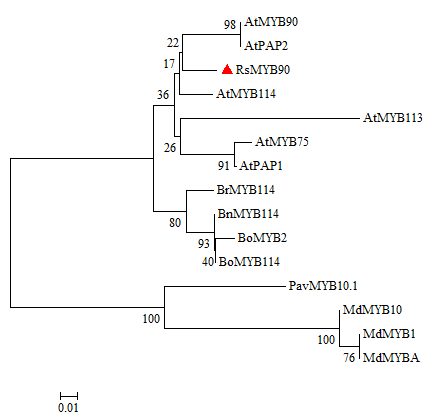
**

**Figure S6.** Phylogenetic tree of RsMYB90 and R2R3 MYBs from other plant species. Numbers at branch nodes represent percent bootstrap values after 1000 replicates, GenBank accession number: AtPAP2(*Arabidopsis thaliana*, OAP17920.1); AtMYB90(*A. thaliana*, [NP_176813.1](https://www.ncbi.nlm.nih.gov/protein/NP_176813.1?report=genbank&log$=protalign&blast_rank=40&RID=MBB2S44X014)); AtMYB113(*A. thaliana*, [NP_176813.1](https://www.ncbi.nlm.nih.gov/protein/NP_176813.1?report=genbank&log$=protalign&blast_rank=40&RID=MBB2S44X014)); AtMYB90(*A. thaliana*, NP_176813.1); AtMYB114(*A. thaliana*, NP_176812.1) AtPAP1(*A. thaliana*, NP_176057.1); AtMYB75(*A. thaliana*, ABB03877.1); BrMYB114(*B. rapa*, NP_001289193.1); BoMYB114(*B. napus*, XP_013590812.1); BnMYB114(*B. oleracea*, XP_013647114.1); BoMYB2(*B. oleracea var. botrytis*, ADP76651.1); PavMYB10.1(*Prunus avium*, KP455683.1); MdMYB10(*Malus domestica*, DQ267896); MdMYB1(*M. domestica*, DQ886414); MdMYA(*M. domestica*, AB242302).

**
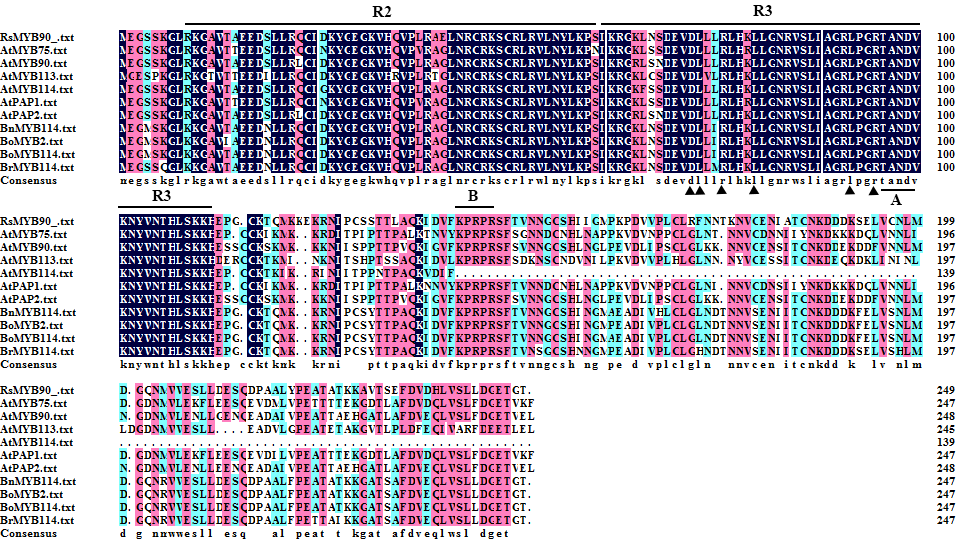
**

**Figure S7.** Protein sequence alignment of RsMYB90 and R2R3 MYB proteins from different species. The R2 and R3 MYB motifs are indicated. Arrowheads indicate all residues needed for a bHLH TF partner, box A is a conserved domain of anthocyanin-regulating MYBs, and box B indicates a conserved C-terminal motif.

**
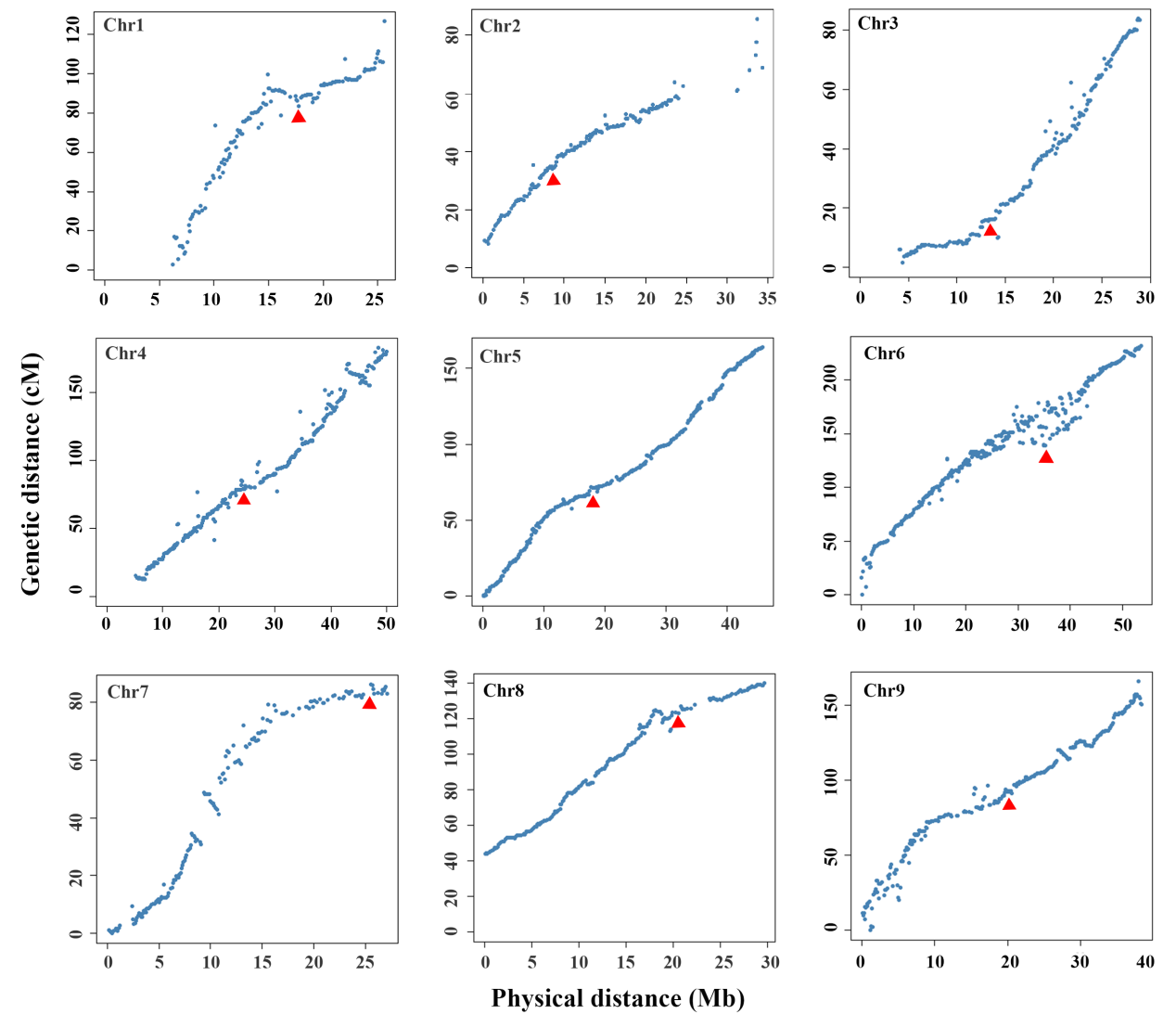
Figure S8.** Genetic versus physical distance maps of 9 chromosomes. X-axis represents the genetic location and Y-axis represents the physical position of the bin markers. The red triangles represent the positions of centromere on each chromosome.





**Figure S9.** The relationship between recombination rate and physical length of the chromosomes.

**
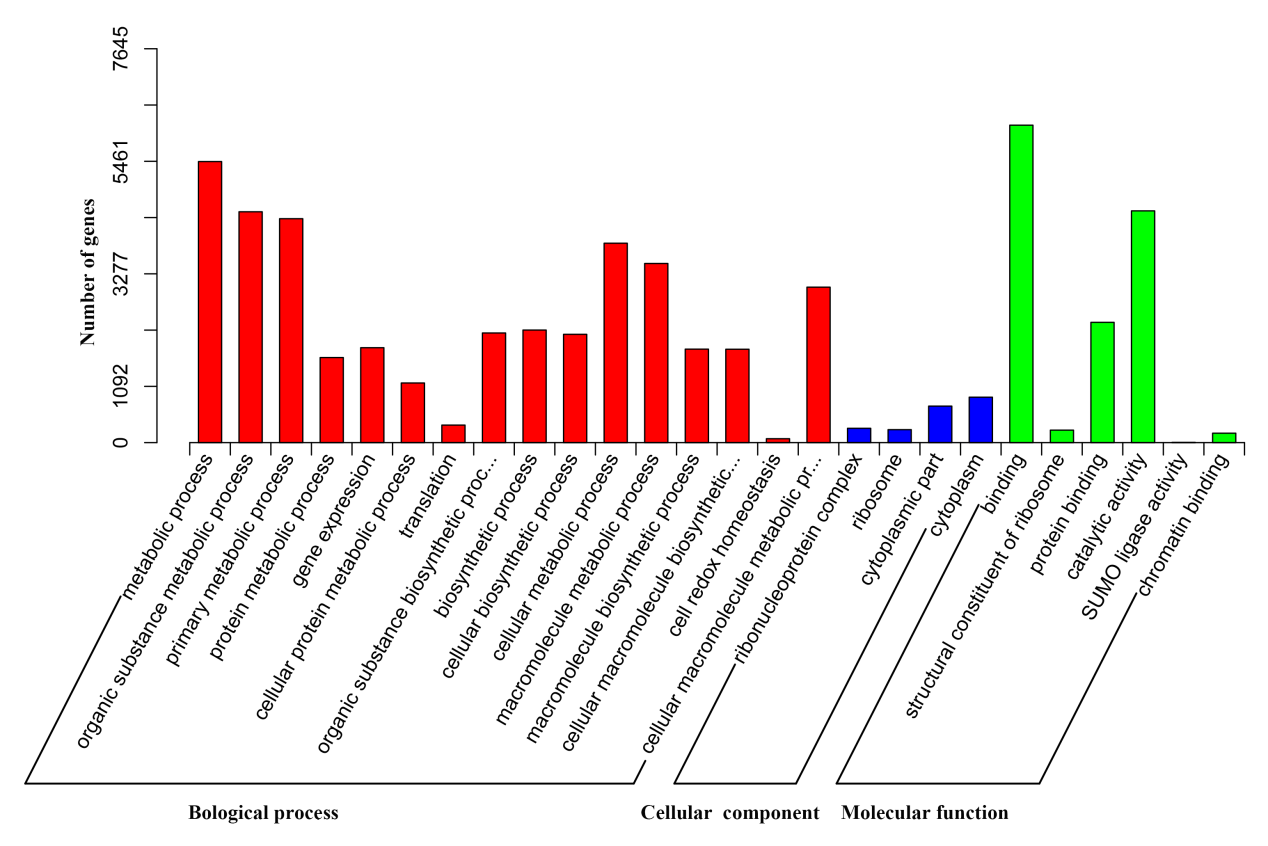
**

**Figure S10.** Gene ontology enrichment of the genes within recombination hot regions.


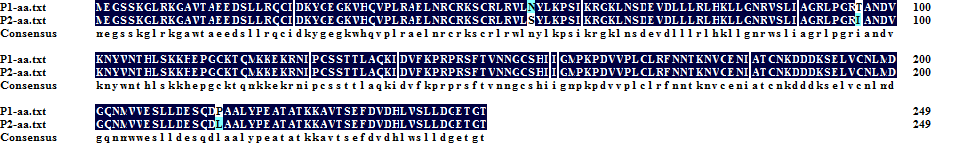

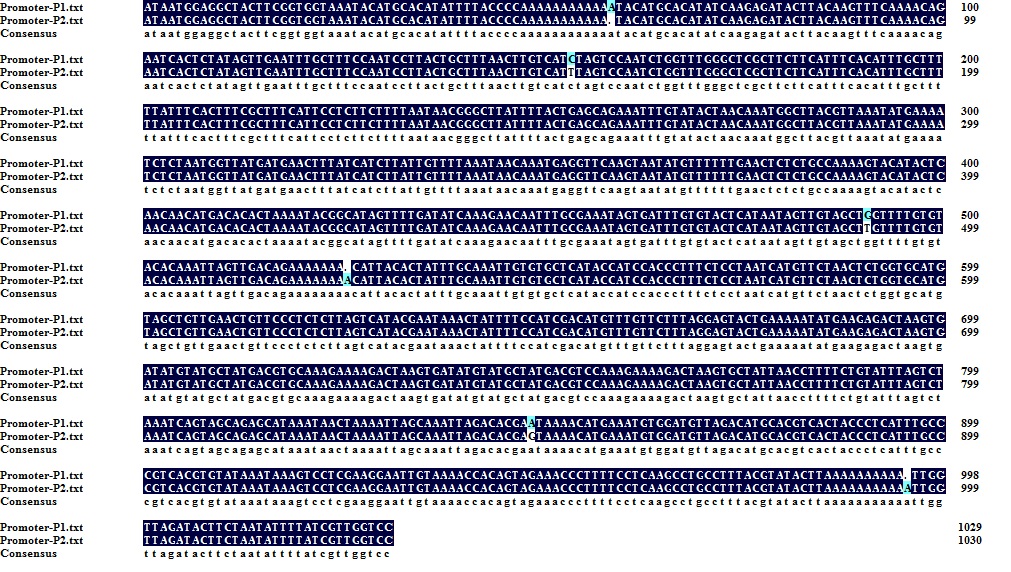


(a)

(b)

**Figure S11.** Amino acid sequence (a) and promoter sequence (b) of *RsMYB90* gene in the two parental lines.
